# Low mean platelet volume is associated with critical limb ischemia in peripheral arterial occlusive disease

**DOI:** 10.1038/s41598-018-25058-8

**Published:** 2018-04-30

**Authors:** Peter Rief, Reinhard B. Raggam, Alexander Avian, Franz Hafner, Mahdi Sareban, Martin Wehrschuetz, Marianne Brodmann, Thomas Gary

**Affiliations:** 1 Division of Vascular Medicine, Department of Internal Medicine, Medical University Graz, Graz, Austria; 2 Institute for Medical Informatics, Statistics and Documentation, Medical University Graz, Graz, Austria; 3 Department of Sports Medicine, Paracelsus Medical Private University Salzburg, Salzburg, Austria; 4 Department of Radiology, Medical University Graz, Graz, Austria

## Abstract

Mean platelet volume (MPV) was recently published as a possible marker of coronary artery disease in patients at high risk for major adverse cardiac events. Because platelets play an important role in atherosclerosis, we examined the relationship between critical limb ischemia (CLI) and MPV in patients with peripheral arterial occlusive disease (PAOD). Our study comprised 2124 PAOD patients. Univariate logistic regression was performed to analyze potential predictors for CLI. Nagelkerke’s R² is reported. Cross validation was performed using the leave-one-out principle. ROC analyses were performed to identify the best cut off value for MPV predicting CLI; to this end, Youden’s index was calculated. For CLI diabetes (p < 0.001, OR 2.44, 95% CI 1.97–3.02), hsCRP (p < 0.001, OR 1.01, 95% CI 1.01–1.01), age (p < 0.001, OR 1.05, 95% CI 1.04–1.06), thrombocytosis (p = 0.025, OR 1.84, 95%CI 1.08–3.14), and MPV (p = 0.003, OR 0.84, 95% CI 0.75–0.94) were significant independent predictors for CLI. ROC analysis (AUC: 0.55, 95% CI 0.52–0.58, p < 0.001) showed ≤10.2 as the best cut off value for MPV to predict CLI. As there is a significant relationship between low MPV and a high risk for CLI in PAOD patients, MPV can be used to identify patients who are likely to develop CLI.

## Introduction

Peripheral arterial occlusive disease (PAOD), a common disorder, must be diagnosed and treated promptly if progression to critical limb ischemia (CLI), with its high mortality and risk of limb amputation, is to be avoided^1,2^. Despite new and improved treatment options developed in recent years, CLI still often leads to amputation and/or death^3,4^.

Platelets play a crucial role in the development of CLI, as there is a high risk for vascular endpoints with a high platelet count. We recently demonstrated that even after adjustment for other well-established vascular risk factors, there was an odds ratio (OR) of 1.9 (95% CI 1.7–2.1) for CLI with a platelet-to-lymphocyte ratio (PLR) of > 150^5^. Our supposition is an interaction of platelets with endothelial cells and leukocytes^6^, with release of inflammatory agents and subsequent adhesion and transmigration of monocytes^7^, whereby the latter are involved in the inflammatory processes that lead to atherosclerosis^8^.

In recent years MPV has been discussed as a potential marker to identify atherosclerosis patients at high risk for an unfavorable outcome. Coronary artery disease (CAD) patients with high MPV seem to be at an especially high risk of death compared to those with low MPV^9^. The respective authors attribute their finding mainly to the denser granules found in larger platelets that seem to express more adhesive receptors, so leading to a more thrombotic state^10^.

As CLI has not yet been included as a vascular endpoint in published studies, and since platelets seem to be substantially involved in vascular disease, we looked into the association of MPV with CLI and other vascular endpoints in our PAOD cohort.

## Methods

Our retrospective analysis included 2124 consecutive PAOD patients seen at our angiological service from 2005 to 2010. Our only inclusion criterion was treatment for PAOD at our department during this time period; there were no exclusion criteria.

The International Review Board, Medical University of Graz, Austria, approved the study (IRB Number 24–506 ex 11/12); as performed, the study conformed completely to all relevant guidelines and regulations. The ethics committee deemed that this retrospective analysis of blinded data required neither written nor verbal consent.

Following diagnosis and grading of PAOD on the basis of clinical examination, ankle brachial index (ABI) and duplex scan at our outpatient clinic, patients were scheduled for inpatient treatment of atherosclerotic disease. The Fontaine classification was used to grade PAOD. CLI was present in PAOD patients with ischemic rest pain and/or skin ulceration/gangrene corresponding to Fontaine classes 3 and 4^11^.

After admission, patients’ records were analysed for cardiovascular risk factors and co-morbidities with a standardized questionnaire. Clinical symptoms were assessed with a physical examination. Blood for laboratory studies was drawn after an overnight fast.

### Statistical analyses

Data are presented as mean and standard deviation (SD) or median and interquartile range (IQR) for continuous data and as a frequency for categorical data. To analyze potential predictors for CLI the following variables were analyzed using univariate binary logistic regression using a logit function: clopidogrel treatment (yes/no), acetylsalicylate treatment (yes/no), diabetes (yes/no), thrombocytosis (yes/no), sex (female/male), hsCRP level, age, and MPV. Significant variables in univariate analyses were selected for multivariate logistic regression. Variables in the final model were selected with a backward stepwise procedure. The decision to remove variables was based on a likelihood-ratio test. Nagelkerke’s R² is reported. For the comparision of the impact of the different significant variables the absolute value of standardized estimates. Cross validation was performed using the leave-one-out principle. A p-value of less than 5% was considered significant. ROC analyses were performed to identify the best cut off value to predict CLI for MPV; to this end, Youden’s index was calculated. Further vascular endpoints tested similarly to CLI were myocardial infarction and stroke. For data analysis SPSS 23.0.0.2 (SPSS Inc, Chicago, IL) and SAS 9.4 (2002–2012 by SAS Institute Inc., Cary, NC, USA) were used.

The datasets analyzed during the study are available from the corresponding author on reasonable request.

## Results

### Patient’s characteristics are given in

Calculations for endpoint CLI: According to univariate analysis, higher age (p < 0.001), higher hsCRP values (p < 0.001), female sex (p < 0.001), presence of diabetes (p < 0.001), no clopidogrel treatment (p = 0.004), no acetylsalicylate treatment (p = 0.003), lower MPV values (p < 0.001), and presence of thrombocytosis (p < 0.001) were associated with an increased risk of CLI Table 1. Multivariate logistic regression analysis (R² = 0.199) revealed that presence of diabetes (p < 0.001, OR: 2.4 95%CI: 2.0–3.0) and thrombocytosis (p = 0.025, OR: 1.84 95%CI: 1.08–3.14), higher hsCRP (p < 0.001, OR: 1.012 95%CI: 1.008–1.014) and age (p < 0.001, OR: 1.05 95%CI: 1.04–1.06) and lower MPV (p = 0.003, OR: 0.84 95%CI: 0.75–0.94) were significant variables associated with CLI (Table 2) with highest absolute values of standardized estimates for age (0.31) hsCRP (0.23) and presence of diabetes (0.23). The area under the ROC curve for the naive prediction model for CLI was 0.746 (95% CI: 0.723–0.769), and 0.742 (95% CI: 0.719–0.765) after cross-validation.

**Table 1:** Patient’s characteristics.

Characteristics*		n = 2124
Age (years), median		69.3 ± 12.0
Sex	male	1266 (59.6%)
	female	858 (40.4%)
BMI (kg/m^2^), median		25 (23–28)
**Vascular risk factors**		
Hypertension	no	393 (18.5%)
	yes	1731 (81.5%)
Diabetes	no	1411 (66.4%)
	yes	713 (33.6%)
Smoking	no	982 (46.2%)
	yes	1142 (53.8%)
**Vascular endpoints**		
Myocardial infarction	no	2032 (95.7%)
	yes	92 (4.3%)
Stroke	no	1956 (92.1%)
	yes	168 (7.9%)
CLI	no	1494 (70.4%)
	yes	630 (29.6%)
**Concomitant disease**		
Heart failure	no	1939 (91.3%)
	yes	185 (8.7)
CAD	no	1378 (64.9%)
	yes	746 (35.1%)
Renal impairment	no	1518 (71.5%)
	yes	608 (28.5%)
Atrial fibrillation	no	1756 (82.7%)
	yes	368 (17.3%)
Cerebrovascular disease	no	666 (31.4%)
	yes	1458 (68.6%)

^*^Results are provided as n (%) or median (±interquartile range)

Abbreviations: BMI, Body Mass Index; CLI, critical limb ischemia; CAD, coronary artery disease.

**Table 2:** Significant predictors for CLI.

Endpoint CLI		mean ± SD median (IQR) n (%)	Univariate Regression		Multivariate Regression		
			sign.	OR (95%CI)	sign.	OR (95%CI)	Absolute value of standardized estimates
Age (years)		69.3 ± 12.0	<0**.001**	1.052 (1.042–1.061)	<0**.001**	1.048 (1.038–1.059)	0.31
hsCRP (mg/L)		5.0 (2.0–13.6)	<0**.001**	1.015 (1.011–1.018)	<0**.001**	1.012 (1.008–1.015)	0.23
Sex	female	858 (40.4%)	<0**.001**	1.4 (1.164–1.684)			
Diabetes mellitus	yes	713 (33.6%)	<0**.001**	2.723 (2.250–3.295)	<0**.001**	2.436 (1.973–3.008)	0.23
Clopidogrel treatment	yes	940 (44.3%)	**0.004**	0.764 (0.634–0.919)			
Acetylsalicylate treatment	yes	1294 (60.9%)	**0.003**	0.758 (0.630–0.913)			
MPV (fl)		10.5 (10.0–11.2)	<0**.001**	0.825 (0.744–0.914)	**0.003**	0.840 (0.750–0.941)	0.09
Thrombocytosis	yes	83 (3.9%)	<0**.001**	2.765 (1.776–4.306)	**0.025**	1.841 (1.078–3.144)	0.06

Abbreviations: CLI, critical limb ischemia; MPV, mean platelet volume; sign., significance, hsCRP, high sensitive c-reactive protein.

Occurrence of myocardial infarction (MCI) and stroke were investigated in the same cohort as described above. According to univariate analysis, higher age (p = 0.007), higher hsCRP values (p = 0.001), presence of diabetes (p = 0.004) and clopidogrel treatment (p = 0.016) were associated with an increased risk of MCI. Multivariate logistic regression analysis (R² = 0.05) revealed that higher age (p = 0.008, OR: 1.03 95%CI: 1.01–1.05), higher hsCRP values (p = 0.005, OR: 1.005 95%CI: 1.002–1.009), presence of diabetes (p = 0.011, OR: 1.75 95%CI: 1.14–2.69) and clopidogrel treatment (p = 0.002, OR: 1.96 95%CI: 1.27–3.04) were significant predictors for MCI (Table 3) with highest absolute values of standardized estimates for age (0.18) and clopidogrel treatment (0.18). The area under the ROC curve for the naive prediction model for MCI was 0.680 (95% CI: 0.624–0.737) after cross-validation. According to univariate analysis, higher hsCRP values (p = 0.002), clopidogrel treatment (p = 0.016) and no acetylsalicylate treatment (p = 0.001) were associated with an increased risk of stroke. Multivariate logistic regression analysis (R² = 0.02) revealed that higher hsCRP values (p = 0.002, OR: 1.005 95%CI: 1.002–1.008), clopidogrel treatment (p = 0.025, OR: 1.453 95%CI: 1.048–2.014) and no acetylsalicylate treatment (p = 0.010, OR: 0.652 95%CI: 0.47–0.90) were significant predictors for stroke with similar absolute values of standardized estimates for all predictors (hsCRP: 0.10; clopidogrel treatment: 0.10; acetylsalicylate treatment: 0.11) (Table 4). The area under the ROC curve for the naive prediction model for stroke was 0.658 (95% CI: 0.600–0.716) after cross-validation.

**Table 3:** Significant predictors for MCI (R² = 0.05).

Endpoint MCI		mean ± SD median (IQR) n (%)	Univariate Regression		Multivariate Regression		
			sign.	OR (95%CI)	sign.	OR (95%CI)	Absolute value of standardized estimates
Age (years)		69.3 ± 12.0	**0.007**	1.026 (1.007–1.046)	**0.008**	1.028 (1.007–1.048)	0.18
hsCRP (mg/L)		5.0 (2.0–13.6)	**0.001**	1.006 (1.002–1.010)	**0.005**	1.005 (1.002–1.009)	0.11
Sex	female	858 (40.4%)		0.818 (0.530–1.264)			
Diabetes mellitus	yes	713 (33.6%)	**0.004**	1.868 (1.228–2.841)	**0.011**	1.747 (1.135–2.689)	0.15
Clopidogrel treatment	yes	940 (44.3%)	**0.016**	1.675 (1.099–2.553)	**0.002**	1.962 (1.268–3.036)	0.18
Acetylsalicylate treatment	yes	1294 (60.9%)	0.195	1.342 (0.860–2.094)			
MPV (fl)		10.5 (10.0–11.2)	0.642	1.056 (0.839–1.330)			
Thrombocytosis	yes	83 (3.9%)	0.744	0.822 (0.255–2.655)			

Abbreviations: MPV, mean platelet volume; sign., significance; MCI, myocardial infarction; hsCRP, high sensitive c-reactive protein.

**Table 4:** Significant predictors for stroke.

Endpoint stroke		mean ± SD median (IQR) n (%)	Univariate Regression		Multivariate Regression		
			sign.	OR (95%CI)	sign.	OR (95%CI)	Absolute value of standardized estimates
Age (years)		69.3 ± 12.0	0.162	1.010 (0.996–1.023)			
hsCRP (mg/L)		5.0 (2.0–13.6)	**0.002**	1.005 (1.002–1.008)	**0.002**	1.005 (1.002–1.008)	0.10
Sex	female	858 (40.4%)	0.760	0.951 (0.689–1.313)			
Diabetes	yes	713 (33.6%)	0.785	1.047 (0.752–1.459)			
Clopidogrel treatment	yes	940 (44.3%)	**0.028**	1.425 (1.039–1.954)	**0.025**	1.453 (1.048–2.014)	0.10
Acetylsalicylate treatment	yes	1294 (60.9%)	**0.001**	0.585 (0.427–0.803)	**0.010**	0.652 (0.471–0.904)	0.11
MPV (fl)		10.5 (10.0–11.2)	0.968	0.996 (0.839–1.184)			
Thrombocytosis	yes	83 (3.9%)	0.552	1.254 (0.594–2.646)			

Abbreviations: MPV, mean platelet volume; sign., significance; hsCRP, high sensitive c-reactive protein.

ROC analysis (AUC: 0.55, 95%CI: 0.52–0.58; p < 0.001) revealed a cut-off of ≤10.2 fL for MPV to best predict CLI (sensitivity: 65%, specificity: 42%, positive predictive value: 71%, negative predictive value: 36%). As MPV was not associated with MI and stroke, ROC analyses were not performed for these endpoints.

## Discussion

### Our study demonstrates a significant association of low MPV with higher occurrence of CLI in a large number of PAOD patients

Our findings are partly in contrast to the literature published in the field of atherosclerosis, mainly evaluating death, MI, and stroke as endpoints. We therefore calculated not only CLI as an endpoint in our data; we also evaluated our PAOD patients for MI and stroke as well. In our cohort neither endpoint, high nor low, was associated with MPV. We know, however, that stroke and MI patients differ from PAOD patients. Usually PAOD patients are older than CAD patients; however, when age was included in our regression analysis, low MPV was still associated with elevated CLI rates.

Recently high MPV was only investigated for mortality in a hemodialysis cohort. In nearly 150 000 patients, high MPV was associated with a higher risk for death and low MPV was associated with a lower death risk, even in multivariate analyses^12^. Possible reasons for the association of high MPV with cardiovascular endpoints were found in the Gutenberg Health Study. MPV was published in this study as a possible marker for platelet activation and arterial stiffness^13^. In this context, recently the white blood cell-to-mean platelet volume ratio (WMR) was found to be associated with a poorer outcome in over 3000 patients with ST-segment elevation myocardial infarction, so that low MPV was associated with a poorer outcome in CAD patients^14^.

Platelets are importantly involved in the progression of atherosclerosis. Current research suggests an interaction between platelets and endothelial cells and leukocytes^6^, with release of inflammatory agents and subsequent adhesion and transmigration of monocytes^7^, whereby the latter are involved in the inflammatory processes that lead to atherosclerosis^8^.

Thrombus formation in PAOD is mainly aggravated after plaque rupture by thrombogenic plaque components that set in motion an avalanche of aggregating platelets and fibrin strand formation, critically triggered by platelet-derived tissue factor (TF) based thrombin generation via feedback activation of coagulation loops^15^.

Recent studies have suggested an association not only of platelets but also of platelet-derived microparticles with arterial thrombi that form and progress when there is frank atherosclerosis or vascular injury^16^. The procoagulant activity of TF, whether bloodborne or derived from plaque, is an important feature of these MP, which accordingly play a major part in hemostasis and thrombosis^17^. This may strongly support our finding since platelets with MPV < 10.2 fL can be considered as “MP” themselves providing enormous enlargement of surface activity and allowing for higher cross-reactivity with TF and receptors for platelet aggregation and subsequent thrombus formation.

Our study has two main drawbacks: the retrospective design and elevated MPV calculated from a single blood sample, as this does not allow assessment of MPV over time.

In a large number of PAOD patients we were, however, able to show for the first time that low MPV is associated with CLI but not with MI and stroke. One possible explanation might be an MP-like pathway initiated by small platelets.
